# A Puzzle

**Published:** 1891-11-07

**Authors:** 


### EDITOR'S LETTER-BOX.

[Our correspondents are reminded that prolixity is a great bar to publication, and that brevity of style and conciseness of statement greatly facilitate early insertion.]

### A PUZZLE.

Sir,—In referring to hospital charity, I beg to propose to your readers a puzzle for solution and give two cases typical of many in this great London. We will call them Mrs. A and Mrs. B.

Mrs. A is the wife of a small tradesman in business for himself. She is suffering from some occult disease, which, however, plainly shows its result upon her outward physiognomy. Upon her application at one of our large general hospitals she is told that her husband being in business should send her to a private doctor. She explains that she has been under her own medical man, but not receiving any benefit, she thought that at the hospital she might receive the benefit of the advice and skill of one or more of the cleverest medical gentlemen of this hospital. But no! business bars the way.

Mrs. B, the wife of a navvy out of work, next applies. She states she is suffering from pains internally caused by a fall downstairs; she is at once admitted, examined, and treated as her injuries require. Now, let us analyse the two cases and see which is the most deserving of charity.

For the sake of argument we will say that A's husband earns two pounds per week; he does not visit a public-house having neither the money to spare nor the time to waste. There are four children schooled, clothed, and kept from the streets; he and his wife dress respectably, pay their way, and keep an aged parent, and belong to a benefit society.

B's husband can earn thirty shillings per week when he likes, but more often than not does not like, and when he does earn any money, both himself and wife squander best part in a public-house; their clothes are ragged, and more often than not, the gift of some sympathetic visiting lady. They have no home in the sense of a home, and their children run the streets to their hearts' content, daily imbibing vice in its most vicious form. He belongs to no benefit society, for as he boasts if he dies why the parish must bury him if his mates won't. Now, Sir, the puzzle to me is this: Where does the charity come in by giving advice, attendance, medicine, &c., and restoring to health Mrs. B, so that she may go on as usual, a burden to the poor struggling ratepayers from her birth until her death, in thus helping one who does not help herself, or in giving a little timely assistance, advice, and medicine to Mrs. A to help her to health so that she may be able to resume her home duties and fight through the battle of life and be a useful help to society that she was before stricken down?—Yours truly,

Conundrum.

[Mrs. B, although the wife of a tradesman, could easily obtain the desired medical relief at a hospital, if she would take the trouble to satisfy the authorities that she was really too poor to pay a private practitioner. The idea that hospital treatment is superior to that of respectable private practitioners is a delusion.—Ed.]

